# Evaluation of the Relationship between the Adenosine Triphosphate (ATP) Bioluminescence Assay and the Presence of *Bacillus anthracis* Spores and Vegetative Cells

**DOI:** 10.3390/ijerph110605708

**Published:** 2014-05-28

**Authors:** Shawn G. Gibbs, Harlan Sayles, Erica M. Colbert, Angela Hewlett, Oleg Chaika, Philip W. Smith

**Affiliations:** 1 Department of Environmental, Agricultural & Occupational Health, College of Public Health, University of Nebraska Medical Center, Omaha, NE 68198, USA; E-Mails: Erica.colbert@douglascounty-ne.gov (E.M.C.); chaikaoleg@yahoo.com (O.C.); 2 Department of Biostatistics, College of Public Health, University of Nebraska Medical Center, Omaha, NE 68198, USA.; E-Mail: hsayles@unmc.edu; 3 Section of Infectious Disease, College of Medicine, University of Nebraska Medical Center, Omaha, NE 68198, USA.; E-Mails: alhewlett@unmc.edu (A.H.); pwsmith@unmc.edu (P.W.S.); 4 Center for Preparedness Education, College of Public Health, University of Nebraska Medical Center, Omaha, NE 68198, USA; 5 Department of Epidemiology, College of Public Health, University of Nebraska Medical Center, Omaha, NE 68198, USA

**Keywords:** ATP measurement, *Bacillus anthracis*, cleaning confirmation, rapid detection

## Abstract

*Background*: The Adenosine triphosphate (ATP) bioluminescence assay was utilized in laboratory evaluations to determine the presence and concentration of vegetative and spore forms of *Bacillus anthracis* Sterne 34F2. *Methods*: Seventeen surfaces from the healthcare environment were selected for evaluation. Surfaces were inoculated with 50 µL of organism suspensions at three concentrations of 10^4^, 10^6^, 10^8^ colony forming units per surface (CFU/surface) of *B. anthracis*. Culture-based methods and ATP based methods were utilized to determine concentrations. *Results*: When all concentrations were evaluated together, a positive correlation between log-adjusted CFU and Relative Light Units (RLU) for endospores and vegetative cells was established. When concentrations were evaluated separately, a significant correlation was not demonstrated. *Conclusions*: This study demonstrated a positive correlation for ATP and culture-based methods for the vegetative cells of *B.* *anthracis*. When evaluating the endospores and combining both metabolic states, the ATP measurements and CFU recovered did not correspond to the initial concentrations on the evaluated surfaces. The results of our study show that the low ATP signal which does not correlate well to the CFU results would not make the ATP measuring devises effective in confirming contamination residual from a bioterrorist event.

## 1. Introduction

*Bacillus anthracis*, a Gram-positive, spore forming, rod shaped bacterium, exists in the environment in two states, as metabolically active vegetative cells or as dormant endospores. In unfavorable conditions, the vegetative cells will undergo sporulation to promote the formation of environmentally stable spores. These resistant spores can persist in the environment for months to years, until favorable growth conditions are met again [1]. Due to this ability to undergo sporulation, and the characteristics of being a mesophilic, facultative anaerobe, *B. anthracis* can be found in soils worldwide [2]. Herbivores become infected from ingesting spores from soil, and humans can become infected from contact with an infected animal. Through this exposure, cutaneous or inhalational anthrax can develop. Recent incidents of anthrax have occurred following exposure to infected cattle. Anthrax was confirmed in an Australian knackery worker exposed to infected cattle [3]. In 2011, nine cases of cutaneous anthrax were reported following villagers butchering, skinning and consuming meat obtained from infected livestock in the Chittoor district, Pradesh, India [4]. Many anthrax cases have been associated with those who make or use musical drums made from natural animal hides [5,6]. The naturally occurring environmental pathway for anthrax transmission and exposure has been well established. 

In addition to the risk of exposure in the natural environment to these bacteria, there is a bioterrorism threat associated with *B. anthracis*. Pathogenic spores were aerosolized at multiple targeted sites, in the 2001 U.S. Postal Service attacks, making the threat of bioterrorism a reality and a major threat to the security of the nation [7]. Bioterrorism and weaponization of *B. anthracis* is a likely public health threat because of the ease of obtaining these bacteria from the natural environment, preparing them to become pathogenic, spreading (aerozolization of endospores) through the environment, and the bacteria’s spores are resistance to heat and sodium hypochlorite (bleach) disinfection [8]. While, *B. anthracis* can be cultured with limited resources, its weaponization is much more difficult [9].

Due to the potential for residual *B. anthracis* from a bioterrorism event it is necessary to find reliable methods for quick assessments of cleanliness of hospitals or bioterrorist target sites. Hospitals are vulnerable to contamination from *B. anthracis* as individuals contaminated from a bioterrorist event or those infected naturally may present to the hospital while still contaminated and prior to knowledge of either their contamination or diagnosis being known to the facility. Thus, they have the potential to contaminate multiple areas within the hospital or other facility they may visit. Currently, microbial contamination is determined via culture-based methodologies which require trained professional staff and can be time consuming expensive, and hazardous. It is important to have a quick method for determining cleanliness, both in the laboratory and the healthcare setting. Based on information gathered from the food industry’s previous twenty years of research with adenosine triphosphate (ATP) bioluminescence [10,11], researchers have begun to use ATP bioluminescence detection methods to determine hospital surfaces’ cleanliness following decontamination [12,13,14]. While ATP bioluminescence could not identify species, it has the potential to show if an organism, such as *B. anthracis*, were present following a known contamination event.

If ATP measurements were demonstrated to be a reliable and accurate estimation of *B. anthracis* contamination on multiple surfaces, the next step would be to factor in potential environmental contamination to then determine if the technology could be used in place of standard microbial plate counts. Thus, reducing the need to perform time-consuming plate counts. Therefore, as a first step this study aimed to determine if the ATP measurements corresponded to the level (concentration in colony forming units (CFU) per surface) of contamination with both vegetative cells and endospores of *B. anthracis* Sterne 34F2 on multiple surfaces. 

## 2. Experimental Section

A laboratory study was conducted to evaluate if an ATP bioluminescence assay, using the 3M™ Clean-Trace™ ATP Surface test (3M Health Care, St. Paul, MN, USA), could be used to determine the presence and relative concentration of vegetative and spore forms of *B. anthracis* Sterne 34F2. 

These evaluations were conducted on multiple surfaces at three concentrations of approximately 10^4^, 10^6^, 10^8^ colony forming units per surface (CFU/surface). Seventeen different surfaces that are common to the healthcare environment were selected for evaluation. Surfaces evaluated in this study were either recovered from surplus hospital equipment or purchased from a home improvement store, and were cut to approximately 100 cm^2^ when possible (Table 1).

**Table 1:** Healthcare related surfaces (coupons) that were evaluated.

Item Description	Surface Area (cm^2^)
Aluminum-sheet metal (Steelworks, Cincinnati, OH, USA)	100
Bed mattress fabric—cut from old hospital mattress from a Hill-Rom Advance 2000 (Hill-Rom Holdings, Inc., Batesville, IN, USA)	100
Bed rail made of Polyvinyl Chloride (PVC)—cut from an old hospital bed from a Hill-Rom Advance 2000	100
Carpet-coronet idolized esteemed berber similar to the weave used in the hospital made of nylone (Coronet, Dalton, GA, USA)	100
Ceramic tile-white (U. S. Ceramic Tile Company part of Roca Tile Group, Miami, FL, USA)	110
Chrome light switch cover—polished material similar to some faucets (Liberty Harware, Winston-Salem, NC, USA)	50
Keyboard (Dell, Round Rock, TX, USA)	50
Lexan polycarbonate sheet (Lowe’s, Omaha, NE, USA)	100
Nickel light switch cover—satin material similar to some faucets (Liberty Harware, Winston-Salem, NC, USA)	100
Paper (Boise White Paper, LLC, Boise, ID, USA)	100
Plastic Acrylic Sheet (Lowe’s, Omaha, NE, USA)	100
Plastic light switch cover (Cooper LightingUnbreakable, Peachtree City, GA, USA)	80
Porcelain tile—rialto Beige (Del Conca, Loudon, TN, USA)	100
PVC-ranch base molding (Royal Molding, Woodbridge, Ontario, Canada)	112
Stainless steel—T304 (Steelworks, Cincinnati, OH, USA)	100
Vinyl flooring—overlook II Sandstone (Armstrong, Lancaster, PA, USA)	100
Wood—stained similar to chair arms-Autumn Maple (Mullican Flooring, Johnson City, TN, USA)	112.5

A number of coupons were kept their original size so the surface area of the coupons varies, but results are always reported per surface. Coupons were cleaned immediately after experimentation by using a 5,000–6,000 ppm sodium hypochlorite soak for 20 min that was pH adjusted to 7 using acetic acid, sterile water rinses, and then autoclaved, when possible, with storage under sterile conditions until evaluation. Quality assurance demonstrated the success of the sterilization process throughout the study. All coupons were inspected prior to use and any coupon showing any signs of material degradation was replaced with a new coupon.

For each evaluation 12 coupons of each surface were utilized with three coupons as the negative ATP control, three as the negative culturable control, three as the ATP exposure, and three as the culturable exposure. Each evaluation was conducted in triplicate for both the vegetative and spore forms of *B. anthracis* and at each of the three predetermined target concentrations (10^4^, 10^6^, 10^8^ CFU/surface). Each of the exposure coupons had 50 microliters (μL) of the organism suspension placed upon its surface to bring the level of organisms per surface up to concentration (10^4^, 10^6^, 10^8^ CFU/surface). This was accomplished by pipetting five drops of 10 μL each of an organism stock solution evenly onto the coupon surface. The coupons were left undisturbed and allowed to dry in a biosafety cabinet for ten minutes prior to being swabbed for analysis with either culture or ATP.

### 2.1. Inoculate Preparation

Pure cultures of *B. anthracis* Sterne 34F2 were utilized to prepare an inoculate suspension of vegetative *B. anthracis* at the desired experimental concentrations. The inoculate was prepared by transferring a colony forming unit from a tryptic soy agar (TSA) plate to a tube of 40 mL of tryptic soy broth (TSB), and allowing it to incubate at 37 °C for at least 24 h or until sufficient growth at 37 °C [15,16]. Multiple tubes were incubated at a time in order to assure the correct concentrations. The tubes were centrifuged for 15 min at 4 °C at 1,000× g on a Beckman TH-4 rotor (Beckman Coulter, Brea, CA, USA) to get a soft pellet of bacterial cells. The supernatant was then decanted and the procedure repeated twice. Then the resulting pellet was resuspended in phosphate buffered saline and combined with other tubes to achieve the appropriate concentration for the stock suspension. The stock solution was evaluated for total count utilizing microscopic and culturable methodology to assure clean vegetative cells [15,16].

The spore suspension was created by spreading the vegetative suspension of *B. anthracis* Sterne 34F2 onto Modified TSA w/5% SBCaCl_2_, MnCl_2_ plates and allowing them to incubate at 37 °C for 48 h, and then an additional 24 h at room temperature [15,16]. The resulting biomass from at least three plates, depending on desired concentration, was suspended into 30 mL sterile distilled water (dH_2_O). This suspension was held at room temperature for 72 h to allow the lysis of vegetative cells. The solution was then centrifuged at 8,000× g for 10 min at 4 °C, washed with dH_2_O, then centrifuged again. The spore suspension was evaluated for viability and observed microscopically to confirm the cleanliness of the suspension and that at least 90% of those were bright refractive endospores. The final supernatant was resuspended in dH_2_O, and adjusted to the desired stock solution concentration [15,16]. The spore suspensions were held at 4°C and utilized within two weeks before a new stock solution was created.

### 2.2. Culturable Microbiological Methods

Throughout the study both culture-based methods and ATP based methods were used to determine the concentrations of *B. anthracis* in suspensions and on an inoculated surfaces. For culture methods a pre-moistened sterile polyester tipped swab (Thermo Fisher Scientific, Waltham, MA, USA) with phosphate buffered saline but no Tween nor inhibitors was swabbed for 30 s against the coupon surfaces (control and experimental). The culture method and the ATP swabs were made as similar as possible. The tip of the swab was removed and placed into 1 mL of sterile phosphate buffered saline (vegetative cells) or Milli-Q water (endospores). Then the Eppendorf tube was vortexed for 30 s. Serial dilution was conducted in triplicate onto tryptic soy agar and incubated for 48 h at 37 °C. The resulting counts were then reported as CFU/surface.

### 2.3. ATP Bioluminescence Methods

ATP based methods were based upon the manufacturer’s recommendation for the 3M™ Clean-Trace™ ATP Surface Test kit, including the 3M™ Clean-Trace™ ATP Surface swabs and 3M™ Clean-Trace™ NGi Luminometer. Like the culture methods the coupon was swabbed for 30 s with the pre-moistened 3M™ Clean-Trace ATP Surface swabs. The ATP swabs were activated according to manufacturer instructions and then the Clean-Trace NGi Luminometer was used to determine the ATP measurement, which were reported digitally as Relative Light Units (RLU).

### 2.4. Quality Control/Quality Assurance

Triplicate evaluation of the organism suspension (CFU and RLU) was conducted both before and after each surface was tested to ensure consistency. Any test that was not within the predetermined limits was disregarded and the evaluation was repeated. Additionally, standard positive and negative control measures were conducted for serial dilution and agar plating.

### 2.5. Statistical Methods

Prior to analysis adjustments were done to remove background noise and to correct for differences in intended *versus* actual suspension concentrations. Each session of lab work included three ATP control measurements and three culture-based control measurements taken prior to and at the conclusion of all work. These control measurements were repeated for each surface used during the session and were taken on surfaces that had just undergone sterilization. Control values were calculated separately for ATP and culture-based measurements. Each control value was calculated as the average of all six culture-based or ATP control measurements collected during that session. These control values were subtracted from all measured experimental values collected during the session to remove the background noise. To correct for session to session variance in suspension concentration, the measurements were then multiplied by a factor equal to the nominal concentration divided by the actual concentration as measured by CFU analysis. These second-level adjustment factors were also calculated on a session by session basis.

Analysis was done using the base 10 logarithms of adjusted values and consisted of using Pearson correlation coefficients to compare ATP and culture-based measurements overall, by organism stage, by suspension concentration, and by surface. Analyses were conducted using SAS/STAT software, Version 9.3 (SAS Institute, Cary, NC, USA) with a p-value greater than 0.05 used to indicate significance.

## 3. Results and Discussion

Throughout the study the target concentrations for CFU/surface were achieved; however, since multiple organism suspensions had to be created throughout the study period it was necessary to standardize. As a result, we recorded both actual and adjusted concentration of RLU and CFU per surface evaluated, which allowed the comparison despite variations within each of the organism suspension (Table 2). In examining Table 2, it is clear that the actual and adjusted concentrations for both CFU and RLU are similar within an organism concentration, but by doing the adjustment it allows for a better comparison across organism concentrations.

**Table 2:** *B. anthracis* Sterne 34F2 (endospore and vegetative cell) concentrations on surfaces *.

Target Concentration CFU/Surface	Variable	Mean	Standard Deviation	Median	Minimum	Maximum
Endospores 10^4^	Actual RLU/Surface	2.15 × 10^1^	1.47 × 10^1^	1.67 × 10^1^	8.00 × 10^0^	6.63 × 10^1^
	Adjusted RLU/Surface	2.40 × 10^1^	1.59 × 10^1^	1.88 × 10^1^	1.09 × 10^1^	6.92 × 10^1^
	Actual CFU/Surface	1.70 × 10^3^	7.61 × 10^2^	1.73 × 10^3^	6.60 × 10^2^	3.20 × 10^3^
	Adjusted CFU/Surface	1.96 × 10^3^	1.02 × 10^3^	2.04 × 10^3^	7.42 × 10^2^	4.42 × 10^3^
Endospores 10^6^	Actual RLU/Surface	2.82 × 10^1^	1.48 × 10^1^	2.70 × 10^1^	9.33 × 10^0^	6.67 × 10^1^
	Adjusted RLU/Surface	2.82 × 10^1^	1.82 × 10^1^	2.70 × 10^1^	4.79 × 10^0^	8.46 × 10^1^
	Actual CFU/Surface	1.96 × 10^5^	1.19 × 10^5^	1.54 × 10^5^	5.93 × 10^4^	5.13 × 10^5^
	Adjusted CFU/Surface	2.21 × 10^5^	1.59 × 10^5^	2.15 × 10^5^	1.17 × 10^4^	5.97 × 10^5^
Endospores 10^8^	Actual RLU/Surface	3.41 × 10^1^	1.31 × 10^1^	3.13 × 10^1^	1.77 × 10^1^	6.13 × 10^1^
	Adjusted RLU/Surface	5.33 × 10^1^	2.30 × 10^1^	4.69 × 10^1^	1.95 × 10^1^	9.84 × 10^1^
	Actual CFU/Surface	1.65 × 10^7^	1.13 × 10^7^	1.28 × 10^7^	4.07 × 10^6^	4.67 × 10^7^
	Adjusted CFU/Surface	2.47 × 10^7^	1.54 × 10^7^	2.11 × 10^7^	6.59 × 10^6^	6.79 × 10^7^
Vegetative Cells 10^4^	Actual RLU/Surface	9.81 × 10^2^	7.55 × 10^2^	1.09 × 10^3^	7.53 × 10^1^	2.44 × 10^3^
	Adjusted RLU/Surface	5.00 × 10^3^	6.24 × 10^3^	1.75 × 10^3^	1.37 × 10^2^	2.09 × 10^4^
	Actual CFU/Surface	1.04 × 10^3^	7.13 × 10^2^	8.47 × 10^2^	2.77 × 10^2^	2.72 × 10^3^
	Adjusted CFU/Surface	2.33 × 10^3^	8.71 × 10^2^	2.26 × 10^3^	9.47 × 10^2^	4.19 × 10^3^
Vegetative Cells 10^6^	Actual RLU/Surface	6.68 × 10^4^	2.95 × 10^4^	7.48 × 10^4^	1.63 × 10^4^	1.17 × 10^5^
	Adjusted RLU/Surface	5.98 × 10^4^	2.20 × 10^4^	5.97 × 10^4^	2.43 × 10^4^	1.05 × 10^5^
	Actual CFU/Surface	3.26 × 10^5^	2.66 × 10^5^	2.74 × 10^5^	4.33 × 10^4^	1.10 × 10^6^
	Adjusted CFU/Surface	2.82 × 10^5^	2.09 × 10^5^	2.11 × 10^5^	6.96 × 10^4^	7.98 × 10^5^
Vegetative Cells 10^8^	Actual RLU/Surface	1.51 × 10^5^	6.76 × 10^4^	1.56 × 10^5^	3.28 × 10^4^	3.13 × 10^5^
	Adjusted RLU/Surface	8.60 × 10^5^	6.16 × 10^5^	6.07 × 10^5^	1.26 × 10^5^	1.99 × 10^6^
	Actual CFU/Surface	4.77 × 10^6^	4.58 × 10^6^	3.30 × 10^6^	5.33 × 10^5^	1.60 × 10^7^
	Adjusted CFU/Surface	2.94 × 10^7^	3.70 × 10^7^	1.32 × 10^7^	1.06 × 10^6^	1.32 × 10^8^

Notes: * Median across the 17 surfaces listed in Table 1. The *n* = 17 in each evaluation.

**Figure 1:**
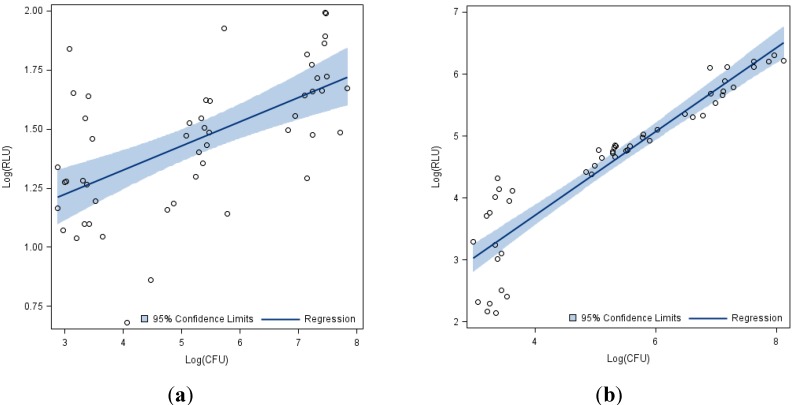
Log adjusted Relative Light Units (RLU) from ATP bioluminescence assay *vs.* Log adjusted Colony Forming Units (CFU) from culture-based methods for *B.* *anthracis* across all 17 surfaces and three concentrations. (**a**) Endospores. (**b**) Vegetative Cells.

The results showed a positive correlation between the log-adjusted CFU and RLU for both endospores (Figure 1a) and vegetative cells (Figure 1b). While this correlation was statistically significant when all three concentrations (10^4^, 10^6^, 10^8^ CFU/Surface) were evaluated together this did not hold when each individual concentration was evaluated (Table 3).

**Table 3:** Correlations between log(RLU) and log(CFU) for *B. anthracis* Sterne 34F2.

Organism	Nominal Slurry Concentration CFU/Surface	N	Pearson Correlation Coefficient log(RLU) *vs.* log(CFU)	*p*-value
Endospores	10^4^	17	−0.085	0.747
	10^6^	17	0.798	<0.001
	10^8^	17	0.340	0.182
	All concentrations	51	0.598	<0.001
Vegetative Cells	10^4^	17	0.271	0.293
	10^6^	17	0.874	<0.001
	10^8^	17	0.886	<0.001
	All concentrations	51	0.925	<0.001
All organisms	All concentrations	102	0.355	<0.001

Seventeen surfaces were evaluated for this study and when all surfaces were evaluated together, there was a positive correlation between the log adjusted CFU and RLU when both endospores and vegetative cells were combined (Table 3). Neither endospores nor vegetative cells showed a statistically significant positive correlation at the 10^4^ concentration. This could be a result of the detection limits for each of the tests, including the manufacturer acknowledgement that 10^3^–10^4^ CFU is at the lower range of the detection limit for the 3M™ Clean-Trace™ ATP Surface Test kit. Additionally, endospores at the 10^8^ concentration did not show a statistically significant correlation which could be the result of the known low levels of ATP in endospores becoming more obvious at higher suspension concentrations [17,18]. The spore form of *B. anthracis* is the form that is used for weaponization [19]. The results of our study show that the low ATP signal which does not correlate well to the CFU results would not make the ATP measuring devices effective in confirming contamination residual from a bioterrorist event. Additionally, the low signal related to the spore form of the organism would likely be lost in the background noise of other ATP and microbial sources that one would encounter in the natural environment.

The correlations for vegetative cells on each individual surface were very strong and statistically significant as were several of the surfaces exposed to spores (Table 4). Nine of the seventeen surfaces evaluated demonstrated a positive and statistically significant correlation between ATP (RLU) and the increasing bacterial spore concentration (CFU) whereas all surfaces demonstrated statistical significance for the vegetative cells (Table 4). However, this trend was lost when combining both the vegetative cells and endospore evaluation. When the vegetative cells and spores were combined and each surface was examined individually, the correlations became much weaker and not significant because the RLU numbers for vegetative cells were consistently much higher (several orders of magnitude) than they were for spores (Table 4).

**Table 4:** Correlations between RLU and CFU on individual surfaces.

Surface Material	Combined Vegetative Cells and Endospores		Vegetative Cells		Endospores	
	Pearson Correlation	*p*-value	Pearson Correlation	*p*-value	Pearson Correlation	*p*-value
Aluminum	0.284	0.254	0.995	<0.001	0.574	0.106
Bed mattress fabric	0.259	0.300	0.970	<0.001	0.843	0.004
Bed rail	0.320	0.196	0.986	<0.001	0.484	0.187
Carpet	0.340	0.168	0.969	<0.001	0.782	0.013
Ceramic tile	0.257	0.303	0.991	<0.001	−0.306	0.424
Chrome light switch cover	0.248	0.321	0.996	<0.001	0.557	0.120
Keyboard	0.319	0.197	0.952	<0.001	0.477	0.195
Lexan polycarbonate	0.516	0.028	0.991	<0.001	0.937	<0.001
Nickel light switch cover	0.421	0.082	0.989	<0.001	0.780	0.013
Paper	0.500	0.035	0.931	<0.001	0.668	0.049
Plastic acrylic sheet	0.308	0.214	0.989	<0.001	0.697	0.037
Plastic light switch cover	0.476	0.054	0.990	<0.001	0.669	0.070
Porcelain tile	0.462	0.054	0.989	<0.001	0.778	0.014
PVC	0.288	0.247	0.985	<0.001	0.727	0.027
Stainless steel	0.528	0.024	0.981	<0.001	0.377	0.318
Vinyl flooring	0.195	0.438	0.998	<0.001	0.844	0.004
Wood-stained	0.231	0.357	0.994	<0.001	−0.006	0.989

Only three of the seventeen surfaces for combined vegetative cells and spores were found to have a statistically significant correlation. This could indicate that we did not have a large enough sample size to determine significance for the other thirteen surfaces or that there are inherent differences between recoveries from each of the surfaces. This could also indicate differences in recovery efficiency across the seventeen surfaces.

It is important to note that stainless steel was one of the surfaces found to have a statistically significant correlation between RLU and CFU, and stainless steel is the carrier surface recommended for several methods conducted by the United States Environmental Protection Agency to evaluate disinfectant efficiency [20]. So, differences in recovery between surfaces could result in differences between laboratory evaluations on stainless steel and disinfectant practices in the field; the surface texture of stainless steel could lend itself to both better disinfection and better recovery than other surfaces commonly found in the healthcare environment. By using stainless steel in surface evaluations and disinfection studies, there could be an inherent inflation of the study results.

This study indicated there was successful recovery and statistically significant relationship between RLU and CFU for vegetative cells on stainless steel but not spores of the same organism. Additionally, stainless steel is not the primary surface found in the hospital room or other environments. This might indicate a need to broaden surface disinfection studies beyond utilizing stainless steel to evaluate disinfection efficiency on a variety of surfaces, including plastics that are common in daily life, in order to get a more complete picture of disinfection as it is likely to take place in hospital environmental cleaning.

There are a number of limitations to our study. First, we only used one device, the 3M™ Clean-Trace™ ATP Surface test, to evaluate the ATP component of our study. Additionally, we only evaluated a limited number of surfaces that do not represent the whole of surface times that would be found in the environment. Also, our study was done under aseptic conditions so there was not the background noise of other ATP and microbial sources that one would encounter in the natural environment. Any future evaluation should consider these limitations.

## 4. Conclusions

The adjusted RLU/surface for the spores never exceeded 10^1^ concentrations, which demonstrated the limited use ATP measuring devices have with spore forming bacteria. RLU/ATP is not a good indication of the level of contamination of spore forming bacteria likely due to the low metabolic activity in the endospore state. When evaluating the presence/absence or concentration of spore forming bacteria, using standard microbiological culture methods are best practices. Therefore, it is our recommendations that ATP measuring devices not be used to try to detect *B. anthracis* spores in the real world environment. This study established a statistically significant and positive relationship between the CFU and RLU of the vegetative form of *B. anthracis*; although not for the spore form. This laboratory study conducted under ideal conditions showed that ATP measuring devices have the potential to be utilized with various surfaces found in the indoor setting to provide an estimation of the level of contamination from vegetative *B. anthracis*; however, further studies must include the background noise of other ATP and microbial sources that one would encounter in the natural environment.
